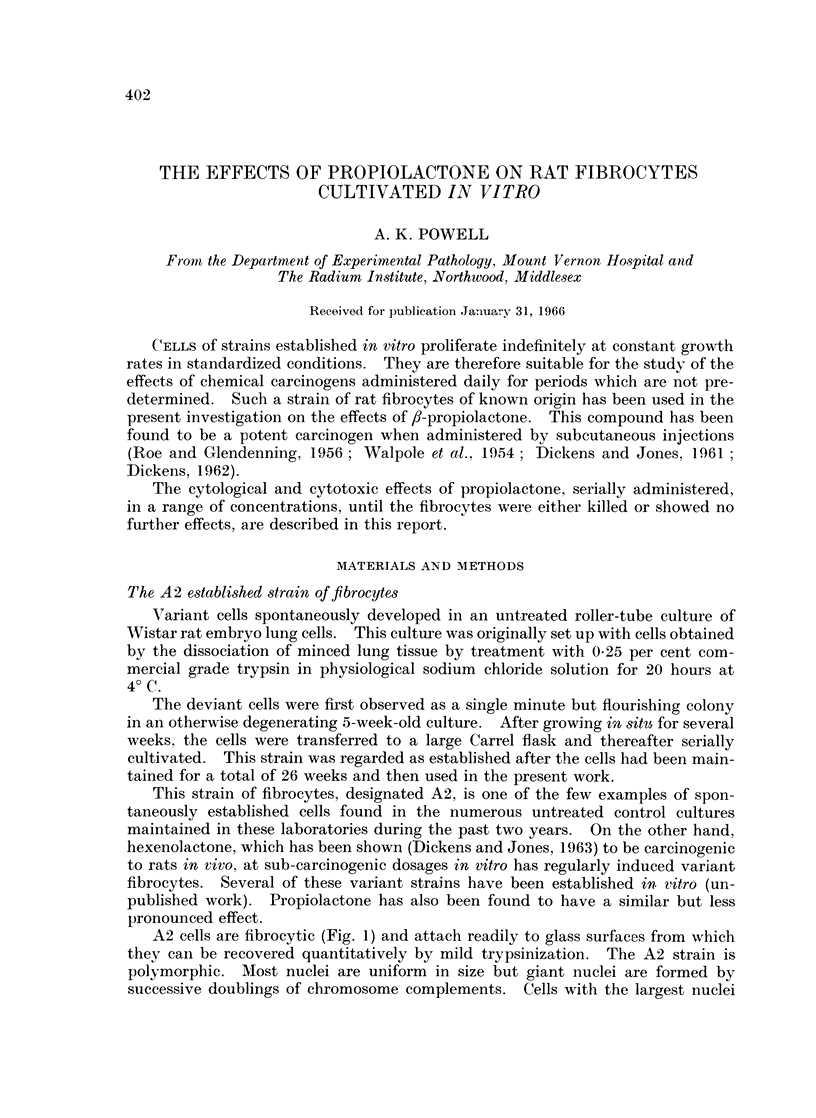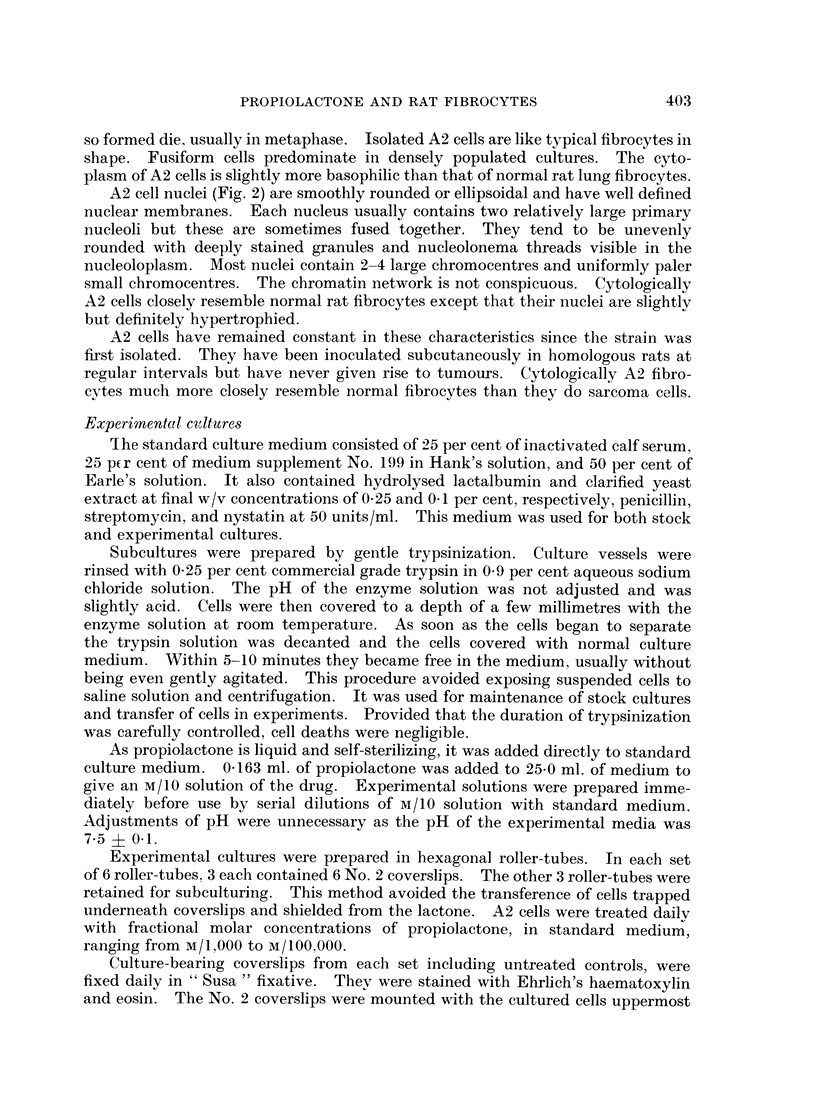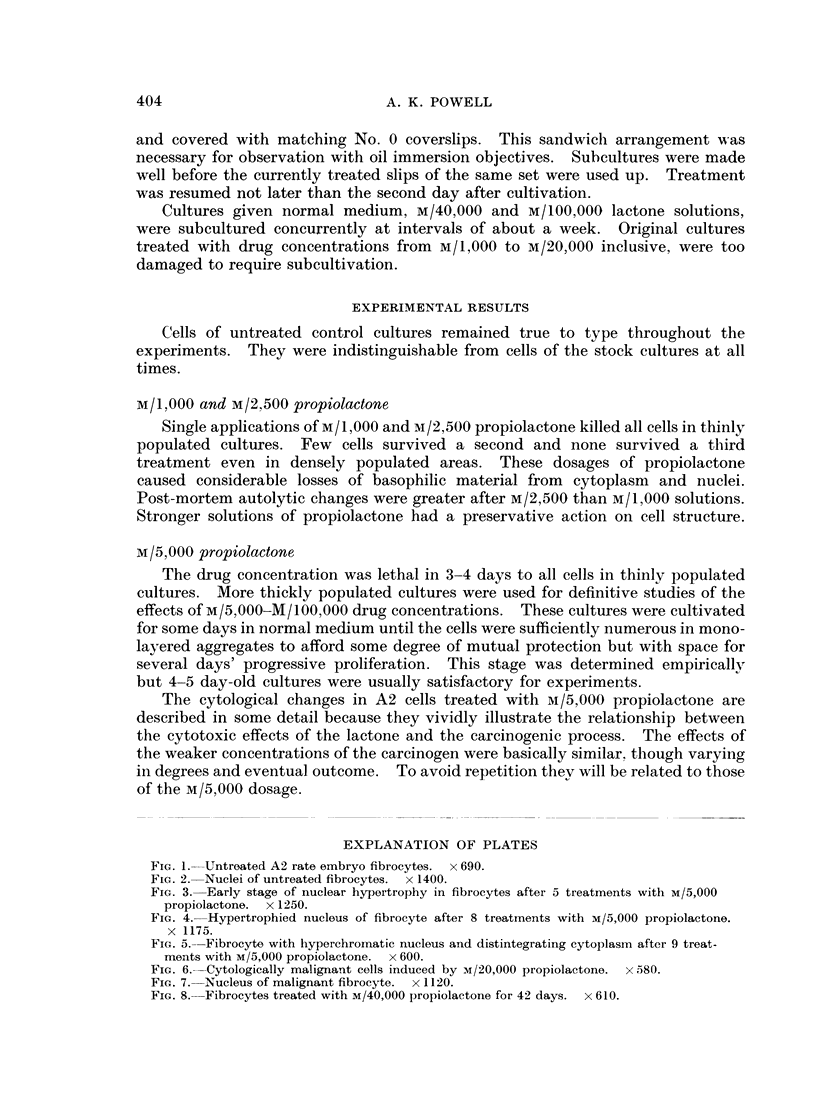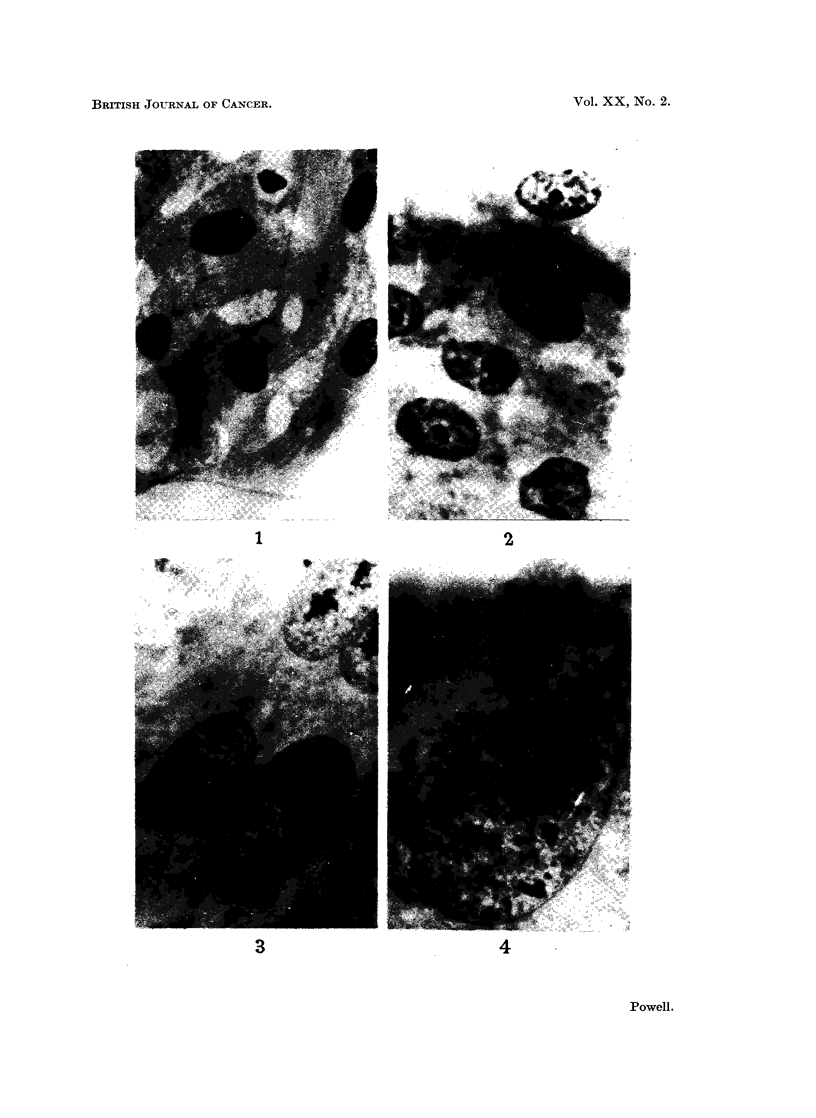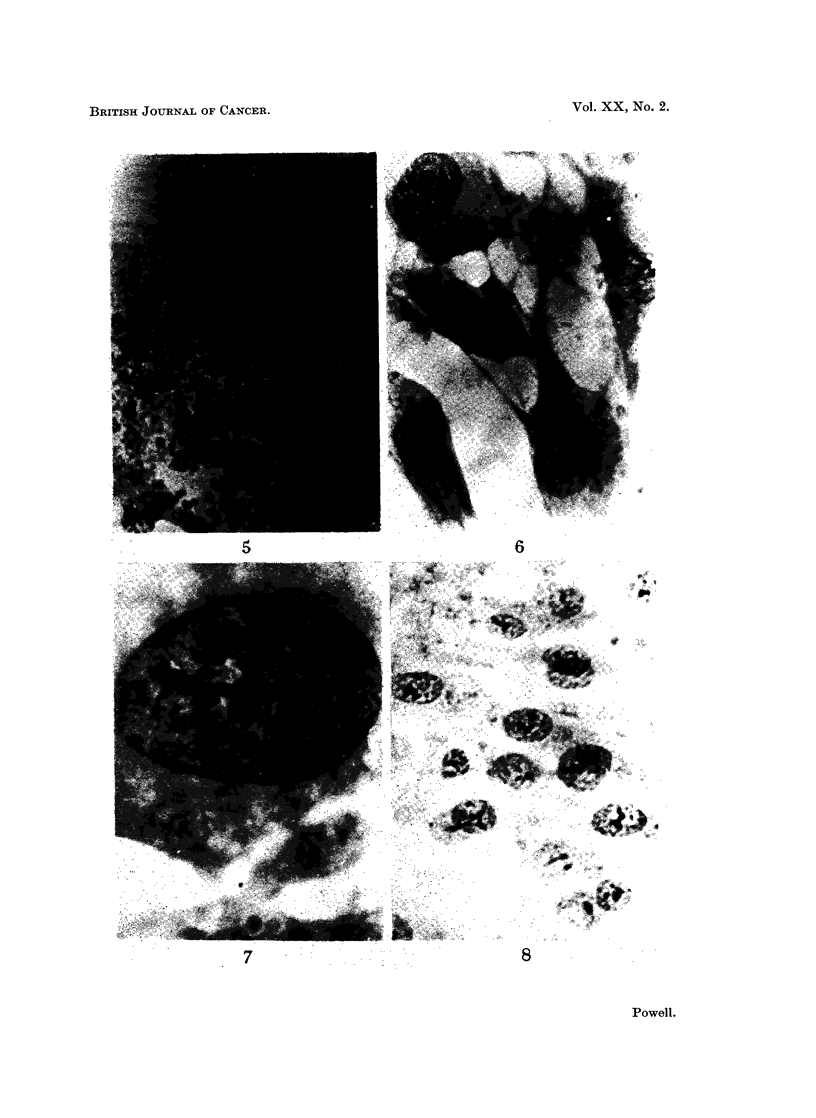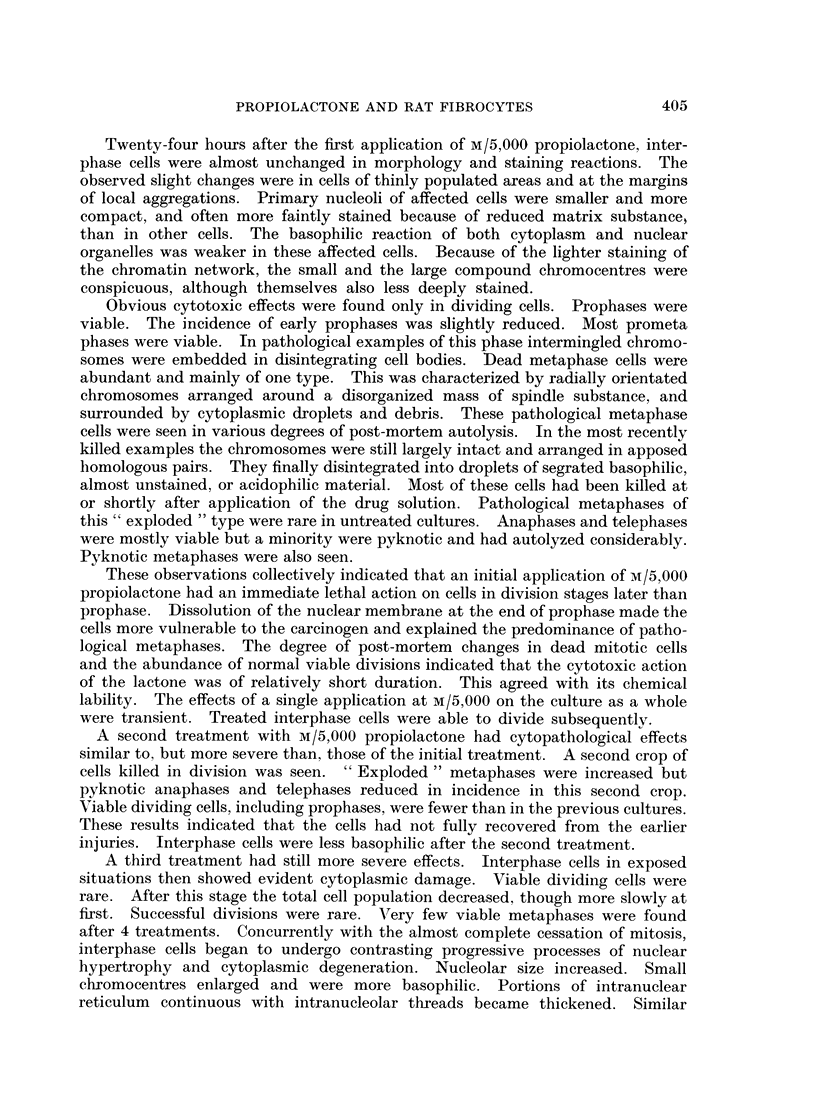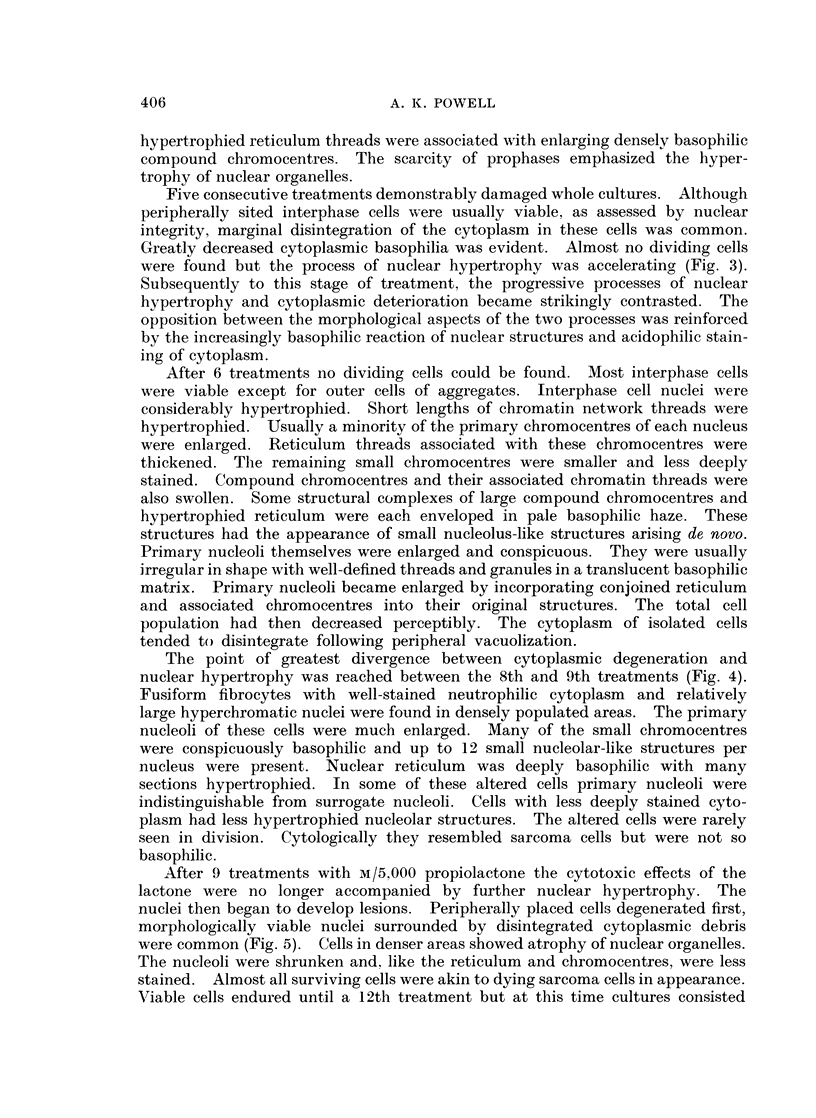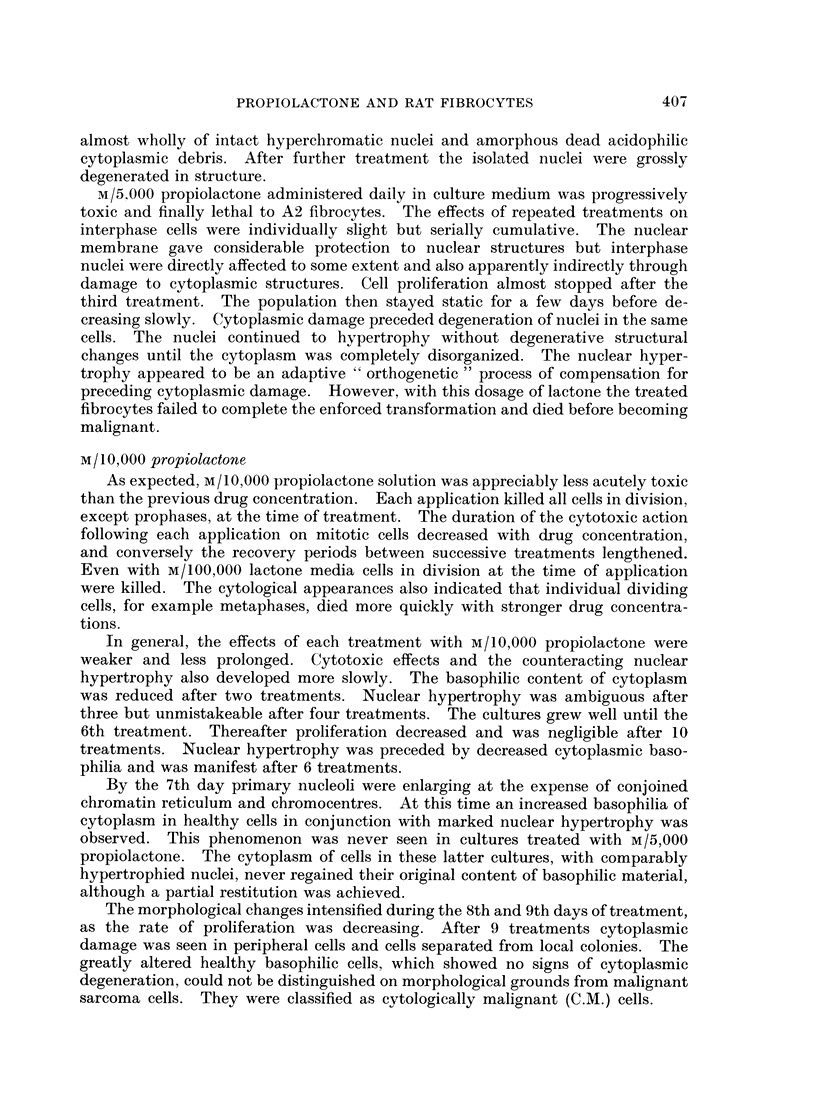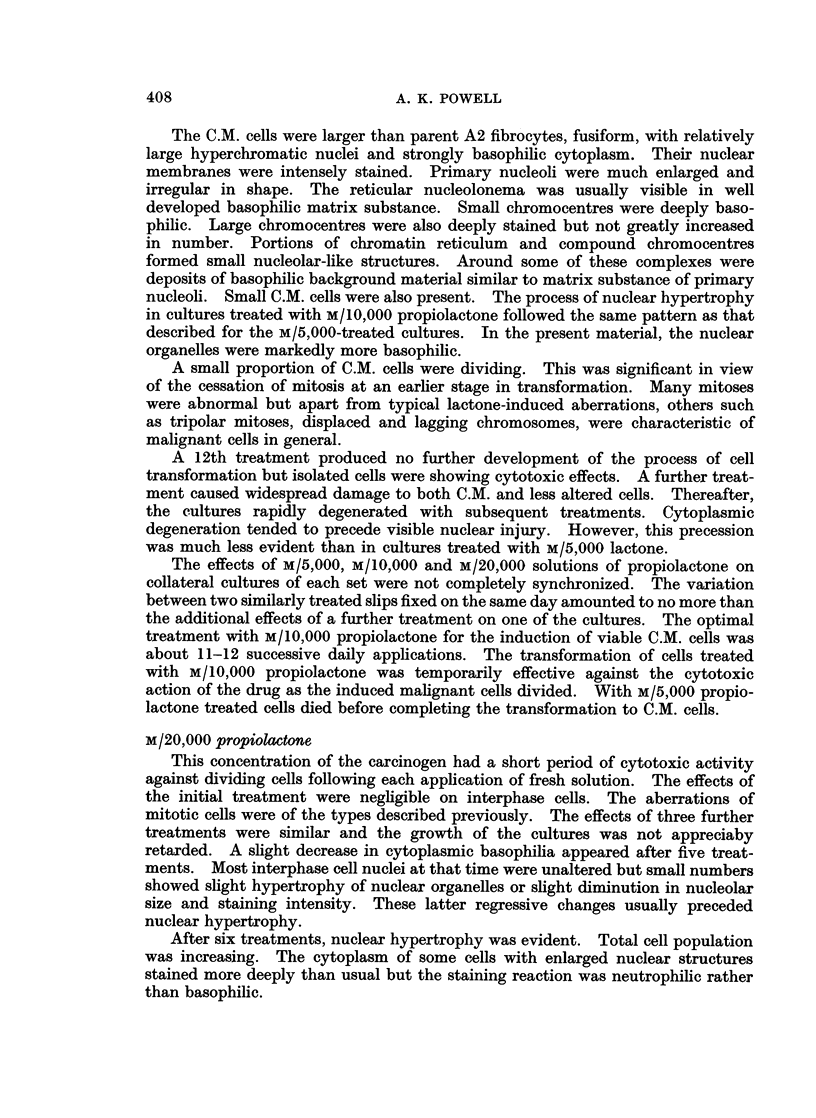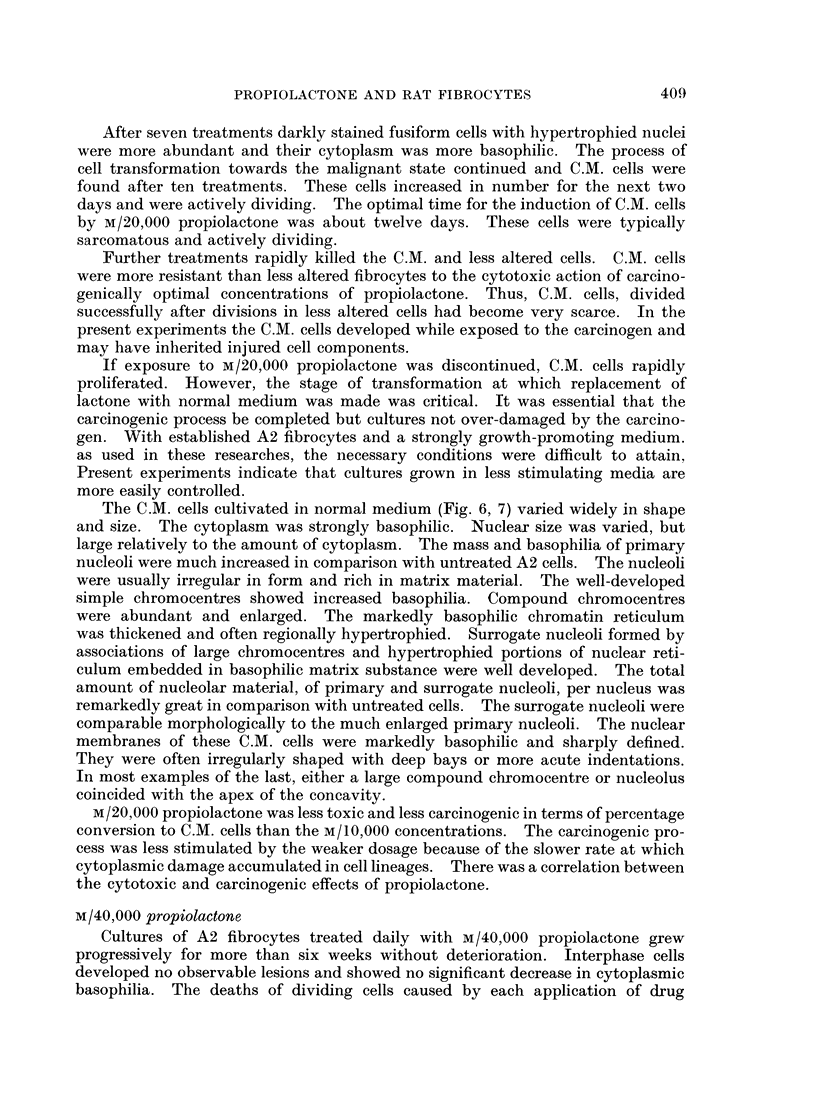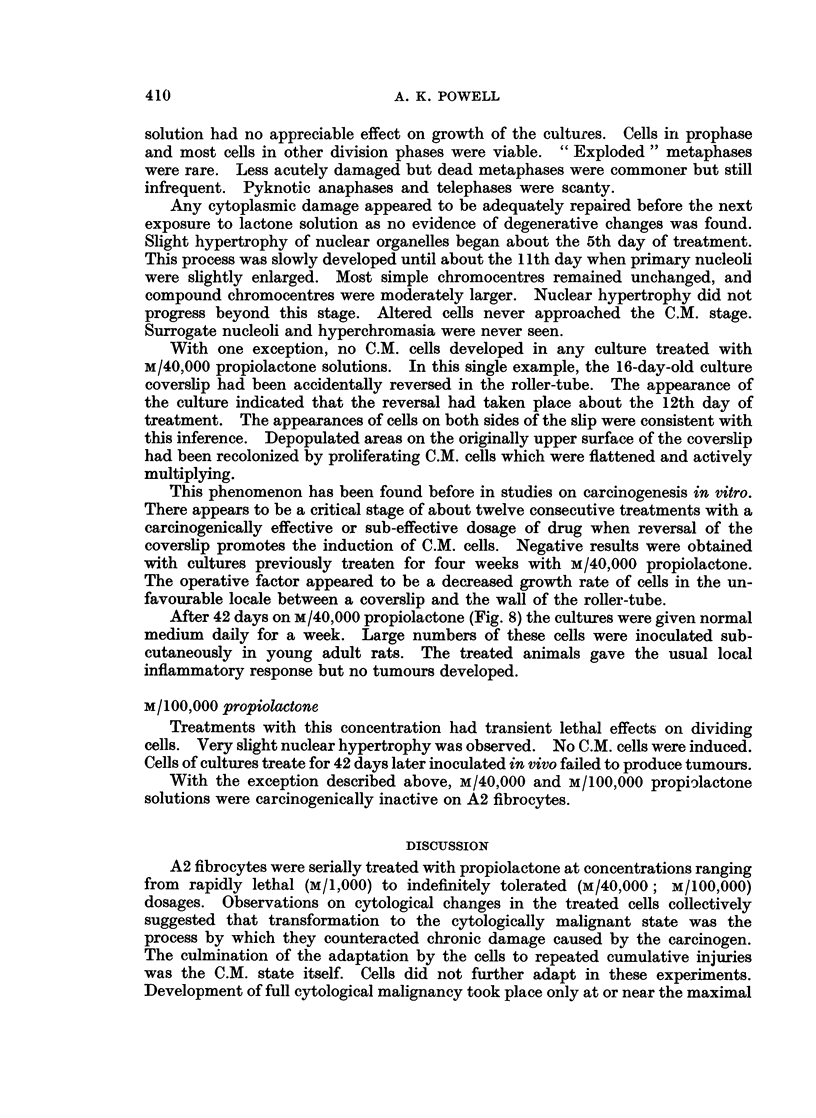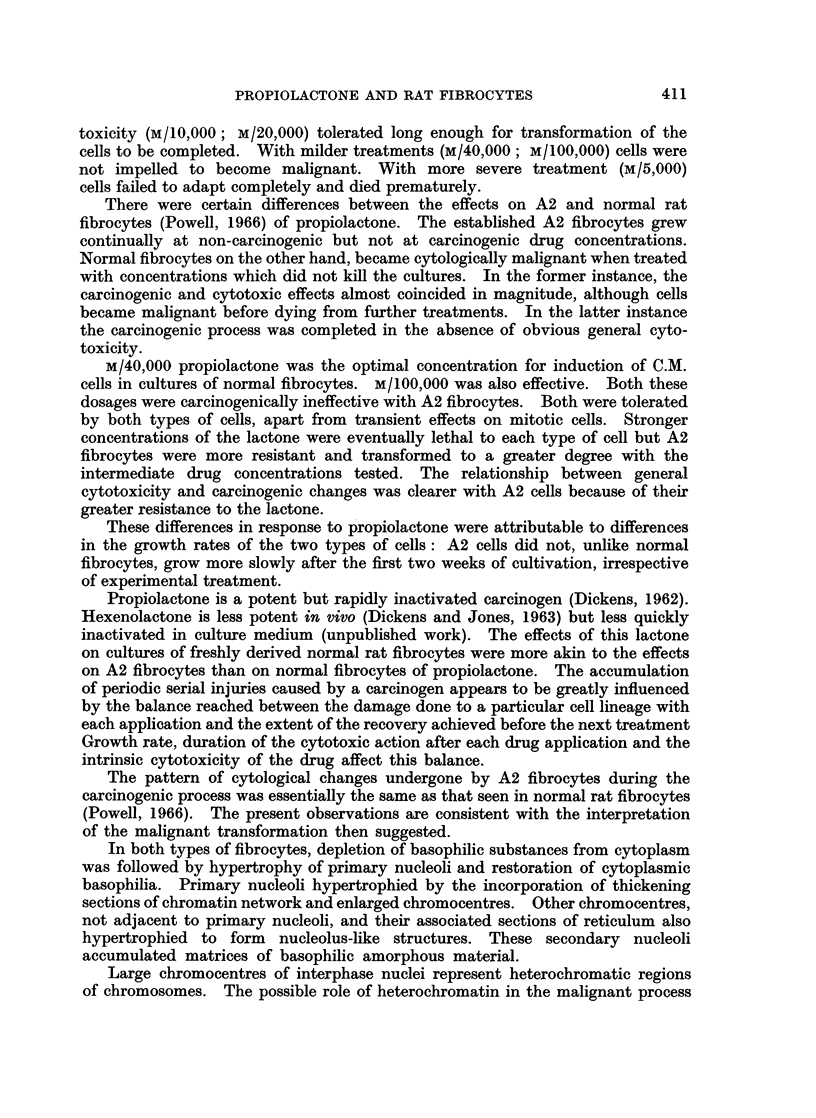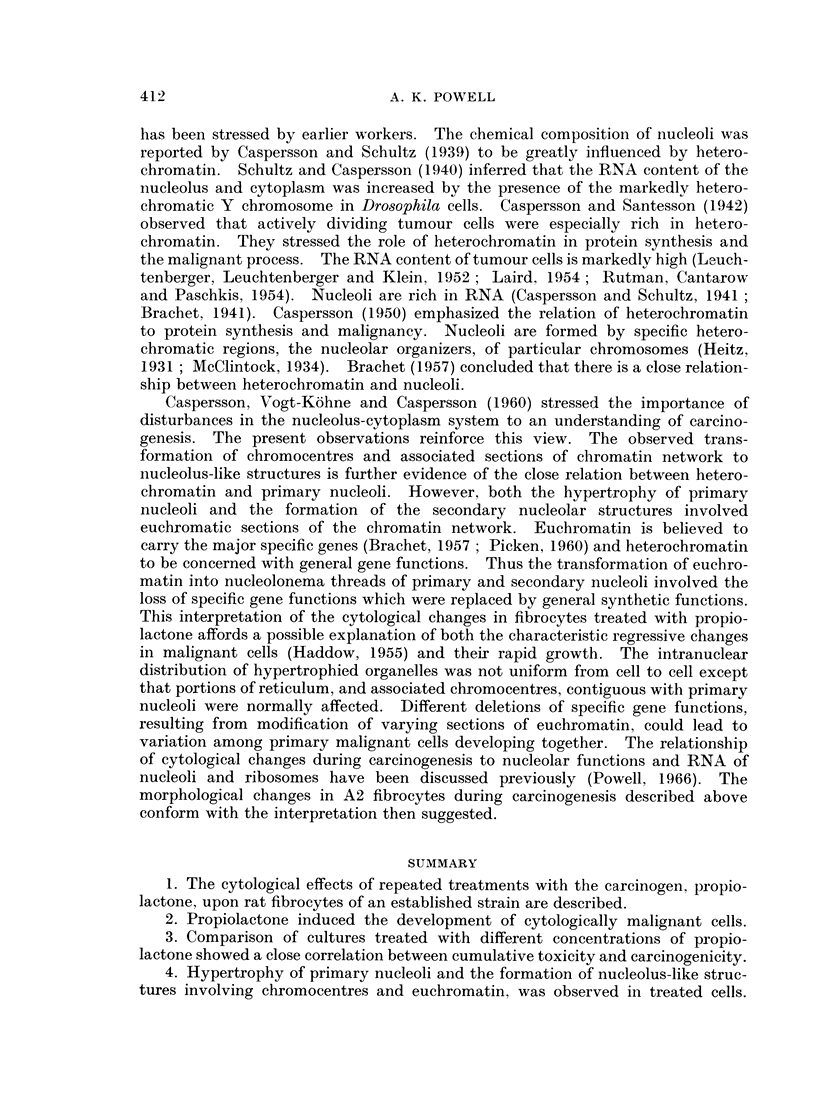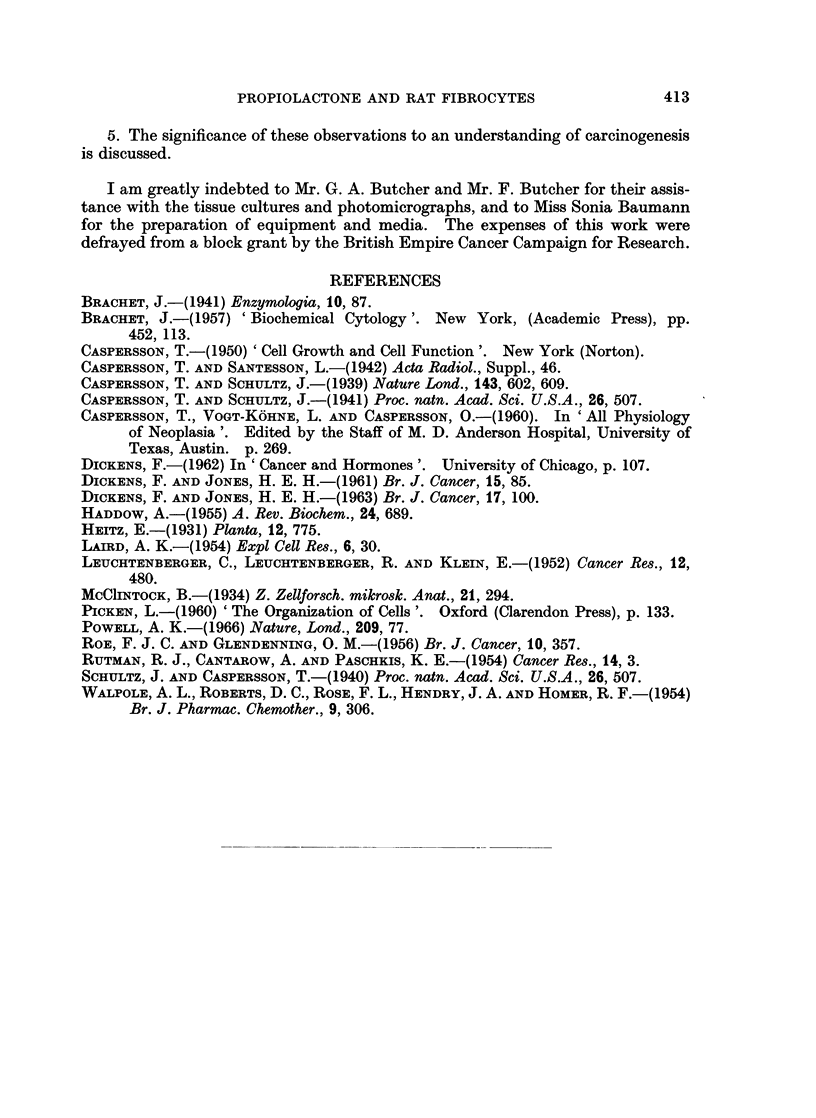# The effects of propiolactone on rat fibrocytes cultivated in vitro.

**DOI:** 10.1038/bjc.1966.48

**Published:** 1966-06

**Authors:** A. K. Powell

## Abstract

**Images:**


### THE EFFECTS OF PROPIOLACTONE ON RAT FIBROCYTES CULTIVATED *IN VITRO*

A. K. POWELL

*From the Department of Experimental Pathology, Mount Vernon Hospital and The Radium Institute, Northwood, Middlesex*

Received for publication January 31, 1966

CELLS of strains established *in vitro* proliferate indefinitely at constant growth rates in standardized conditions. They are therefore suitable for the study of the effects of chemical carcinogens administered daily for periods which are not predetermined. Such a strain of rat fibrocytes of known origin has been used in the present investigation on the effects of $\beta$-propiolactone. This compound has been found to be a potent carcinogen when administered by subcutaneous injections (Roe and Glendenning, 1956 ; Walpole *et al.*, 1954 ; Dickens and Jones, 1961 ; Dickens, 1962).

The cytological and cytotoxic effects of propiolactone, serially administered, in a range of concentrations, until the fibrocytes were either killed or showed no further effects, are described in this report.

### MATERIALS AND METHODS

### *The A2 established strain of fibrocytes*

Variant cells spontaneously developed in an untreated roller-tube culture of Wistar rat embryo lung cells. This culture was originally set up with cells obtained by the dissociation of minced lung tissue by treatment with 0·25 per cent commercial grade trypsin in physiological sodium chloride solution for 20 hours at 4° C.

The deviant cells were first observed as a single minute but flourishing colony in an otherwise degenerating 5-week-old culture. After growing *in situ* for several weeks, the cells were transferred to a large Carrel flask and thereafter serially cultivated. This strain was regarded as established after the cells had been maintained for a total of 26 weeks and then used in the present work.

This strain of fibrocytes, designated A2, is one of the few examples of spontaneously established cells found in the numerous untreated control cultures maintained in these laboratories during the past two years. On the other hand, hexenolactone, which has been shown (Dickens and Jones, 1963) to be carcinogenic to rats *in vivo*, at sub-carcinogenic dosages *in vitro* has regularly induced variant fibrocytes. Several of these variant strains have been established *in vitro* (unpublished work). Propiolactone has also been found to have a similar but less pronounced effect.

A2 cells are fibrocytic (Fig. 1) and attach readily to glass surfaces from which they can be recovered quantitatively by mild trypsinization. The A2 strain is polymorphic. Most nuclei are uniform in size but giant nuclei are formed by successive doublings of chromosome complements. Cells with the largest nuclei so formed die, usually in metaphase. Isolated A2 cells are like typical fibrocytes in shape. Fusiform cells predominate in densely populated cultures. The cytoplasm of A2 cells is slightly more basophilic than that of normal rat lung fibrocytes.

A2 cell nuclei (Fig. 2) are smoothly rounded or ellipsoidal and have well defined nuclear membranes. Each nucleus usually contains two relatively large primary nucleoli but these are sometimes fused together. They tend to be unevenly rounded with deeply stained granules and nucleolonema threads visible in the nucleoloplasm. Most nuclei contain 2–4 large chromocentres and uniformly paler small chromocentres. The chromatin network is not conspicuous. Cytologically A2 cells closely resemble normal rat fibrocytes except that their nuclei are slightly but definitely hypertrophied.

A2 cells have remained constant in these characteristics since the strain was first isolated. They have been inoculated subcutaneously in homologous rats at regular intervals but have never given rise to tumours. Cytologically A2 fibrocytes much more closely resemble normal fibrocytes than they do sarcoma cells.

*Experimental cultures*

The standard culture medium consisted of 25 per cent of inactivated calf serum, 25 per cent of medium supplement No. 199 in Hank's solution, and 50 per cent of Earle's solution. It also contained hydrolysed lactalbumin and clarified yeast extract at final w/v concentrations of 0·25 and 0·1 per cent, respectively, penicillin, streptomycin, and nystatin at 50 units/ml. This medium was used for both stock and experimental cultures.

Subcultures were prepared by gentle trypsinization. Culture vessels were rinsed with 0·25 per cent commercial grade trypsin in 0·9 per cent aqueous sodium chloride solution. The pH of the enzyme solution was not adjusted and was slightly acid. Cells were then covered to a depth of a few millimetres with the enzyme solution at room temperature. As soon as the cells began to separate the trypsin solution was decanted and the cells covered with normal culture medium. Within 5–10 minutes they became free in the medium, usually without being even gently agitated. This procedure avoided exposing suspended cells to saline solution and centrifugation. It was used for maintenance of stock cultures and transfer of cells in experiments. Provided that the duration of trypsinization was carefully controlled, cell deaths were negligible.

As propiolactone is liquid and self-sterilizing, it was added directly to standard culture medium. 0·163 ml. of propiolactone was added to 25·0 ml. of medium to give an M/10 solution of the drug. Experimental solutions were prepared immediately before use by serial dilutions of M/10 solution with standard medium. Adjustments of pH were unnecessary as the pH of the experimental media was 7·5 ± 0·1.

Experimental cultures were prepared in hexagonal roller-tubes. In each set of 6 roller-tubes, 3 each contained 6 No. 2 coverslips. The other 3 roller-tubes were retained for subculturing. This method avoided the transference of cells trapped underneath coverslips and shielded from the lactone. A2 cells were treated daily with fractional molar concentrations of propiolactone, in standard medium, ranging from M/1,000 to M/100,000.

Culture-bearing coverslips from each set including untreated controls, were fixed daily in " Susa " fixative. They were stained with Ehrlich's haematoxylin and eosin. The No. 2 coverslips were mounted with the cultured cells uppermost and covered with matching No. 0 coverslips. This sandwich arrangement was necessary for observation with oil immersion objectives. Subcultures were made well before the currently treated slips of the same set were used up. Treatment was resumed not later than the second day after cultivation.

Cultures given normal medium, M/40,000 and M/100,000 lactone solutions, were subcultured concurrently at intervals of about a week. Original cultures treated with drug concentrations from M/1,000 to M/20,000 inclusive, were too damaged to require subcultivation.

### EXPERIMENTAL RESULTS

Cells of untreated control cultures remained true to type throughout the experiments. They were indistinguishable from cells of the stock cultures at all times.

### M/1,000 *and* M/2,500 *propiolactone*

Single applications of M/1,000 and M/2,500 propiolactone killed all cells in thinly populated cultures. Few cells survived a second and none survived a third treatment even in densely populated areas. These dosages of propiolactone caused considerable losses of basophilic material from cytoplasm and nuclei. Post-mortem autolytic changes were greater after M/2,500 than M/1,000 solutions. Stronger solutions of propiolactone had a preservative action on cell structure.

### M/5,000 *propiolactone*

The drug concentration was lethal in 3–4 days to all cells in thinly populated cultures. More thickly populated cultures were used for definitive studies of the effects of M/5,000–M/100,000 drug concentrations. These cultures were cultivated for some days in normal medium until the cells were sufficiently numerous in monolayered aggregates to afford some degree of mutual protection but with space for several days' progressive proliferation. This stage was determined empirically but 4–5 day-old cultures were usually satisfactory for experiments.

The cytological changes in A2 cells treated with M/5,000 propiolactone are described in some detail because they vividly illustrate the relationship between the cytotoxic effects of the lactone and the carcinogenic process. The effects of the weaker concentrations of the carcinogen were basically similar, though varying in degrees and eventual outcome. To avoid repetition they will be related to those of the M/5,000 dosage.

---

### EXPLANATION OF PLATES

FIG. 1.—Untreated A2 rate embryo fibrocytes. × 690.
FIG. 2.—Nuclei of untreated fibrocytes. × 1400.
FIG. 3.—Early stage of nuclear hypertrophy in fibrocytes after 5 treatments with M/5,000 propiolactone. × 1250.
FIG. 4.—Hypertrophied nucleus of fibrocyte after 8 treatments with M/5,000 propiolactone. × 1175.
FIG. 5.—Fibrocyte with hyperchromatic nucleus and distintegrating cytoplasm after 9 treatments with M/5,000 propiolactone. × 600.
FIG. 6.—Cytologically malignant cells induced by M/20,000 propiolactone. × 580.
FIG. 7.—Nucleus of malignant fibrocyte. × 1120.
FIG. 8.—Fibrocytes treated with M/40,000 propiolactone for 42 days. × 610.

1

2

3

4

Powell.

5

6

7

8

Powell.

Twenty-four hours after the first application of M/5,000 propiolactone, interphase cells were almost unchanged in morphology and staining reactions. The observed slight changes were in cells of thinly populated areas and at the margins of local aggregations. Primary nucleoli of affected cells were smaller and more compact, and often more faintly stained because of reduced matrix substance, than in other cells. The basophilic reaction of both cytoplasm and nuclear organelles was weaker in these affected cells. Because of the lighter staining of the chromatin network, the small and the large compound chromocentres were conspicuous, although themselves also less deeply stained.

Obvious cytotoxic effects were found only in dividing cells. Prophases were viable. The incidence of early prophases was slightly reduced. Most prometa phases were viable. In pathological examples of this phase intermingled chromosomes were embedded in disintegrating cell bodies. Dead metaphase cells were abundant and mainly of one type. This was characterized by radially orientated chromosomes arranged around a disorganized mass of spindle substance, and surrounded by cytoplasmic droplets and debris. These pathological metaphase cells were seen in various degrees of post-mortem autolysis. In the most recently killed examples the chromosomes were still largely intact and arranged in apposed homologous pairs. They finally disintegrated into droplets of segrated basophilic, almost unstained, or acidophilic material. Most of these cells had been killed at or shortly after application of the drug solution. Pathological metaphases of this " exploded " type were rare in untreated cultures. Anaphases and telephases were mostly viable but a minority were pyknotic and had autolyzed considerably. Pyknotic metaphases were also seen.

These observations collectively indicated that an initial application of M/5,000 propiolactone had an immediate lethal action on cells in division stages later than prophase. Dissolution of the nuclear membrane at the end of prophase made the cells more vulnerable to the carcinogen and explained the predominance of pathological metaphases. The degree of post-mortem changes in dead mitotic cells and the abundance of normal viable divisions indicated that the cytotoxic action of the lactone was of relatively short duration. This agreed with its chemical lability. The effects of a single application at M/5,000 on the culture as a whole were transient. Treated interphase cells were able to divide subsequently.

A second treatment with M/5,000 propiolactone had cytopathological effects similar to, but more severe than, those of the initial treatment. A second crop of cells killed in division was seen. " Exploded " metaphases were increased but pyknotic anaphases and telephases reduced in incidence in this second crop. Viable dividing cells, including prophases, were fewer than in the previous cultures. These results indicated that the cells had not fully recovered from the earlier injuries. Interphase cells were less basophilic after the second treatment.

A third treatment had still more severe effects. Interphase cells in exposed situations then showed evident cytoplasmic damage. Viable dividing cells were rare. After this stage the total cell population decreased, though more slowly at first. Successful divisions were rare. Very few viable metaphases were found after 4 treatments. Concurrently with the almost complete cessation of mitosis, interphase cells began to undergo contrasting progressive processes of nuclear hypertrophy and cytoplasmic degeneration. Nucleolar size increased. Small chromocentres enlarged and were more basophilic. Portions of intranuclear reticulum continuous with intranucleolar threads became thickened. Similar hypertrophied reticulum threads were associated with enlarging densely basophilic compound chromocentres. The scarcity of prophases emphasized the hypertrophy of nuclear organelles.

Five consecutive treatments demonstrably damaged whole cultures. Although peripherally sited interphase cells were usually viable, as assessed by nuclear integrity, marginal disintegration of the cytoplasm in these cells was common. Greatly decreased cytoplasmic basophilia was evident. Almost no dividing cells were found but the process of nuclear hypertrophy was accelerating (Fig. 3). Subsequently to this stage of treatment, the progressive processes of nuclear hypertrophy and cytoplasmic deterioration became strikingly contrasted. The opposition between the morphological aspects of the two processes was reinforced by the increasingly basophilic reaction of nuclear structures and acidophilic staining of cytoplasm.

After 6 treatments no dividing cells could be found. Most interphase cells were viable except for outer cells of aggregates. Interphase cell nuclei were considerably hypertrophied. Short lengths of chromatin network threads were hypertrophied. Usually a minority of the primary chromocentres of each nucleus were enlarged. Reticulum threads associated with these chromocentres were thickened. The remaining small chromocentres were smaller and less deeply stained. Compound chromocentres and their associated chromatin threads were also swollen. Some structural complexes of large compound chromocentres and hypertrophied reticulum were each enveloped in pale basophilic haze. These structures had the appearance of small nucleolus-like structures arising *de novo*. Primary nucleoli themselves were enlarged and conspicuous. They were usually irregular in shape with well-defined threads and granules in a translucent basophilic matrix. Primary nucleoli became enlarged by incorporating conjoined reticulum and associated chromocentres into their original structures. The total cell population had then decreased perceptibly. The cytoplasm of isolated cells tended to disintegrate following peripheral vacuolization.

The point of greatest divergence between cytoplasmic degeneration and nuclear hypertrophy was reached between the 8th and 9th treatments (Fig. 4). Fusiform fibrocytes with well-stained neutrophilic cytoplasm and relatively large hyperchromatic nuclei were found in densely populated areas. The primary nucleoli of these cells were much enlarged. Many of the small chromocentres were conspicuously basophilic and up to 12 small nucleolar-like structures per nucleus were present. Nuclear reticulum was deeply basophilic with many sections hypertrophied. In some of these altered cells primary nucleoli were indistinguishable from surrogate nucleoli. Cells with less deeply stained cytoplasm had less hypertrophied nucleolar structures. The altered cells were rarely seen in division. Cytologically they resembled sarcoma cells but were not so basophilic.

After 9 treatments with M/5,000 propiolactone the cytotoxic effects of the lactone were no longer accompanied by further nuclear hypertrophy. The nuclei then began to develop lesions. Peripherally placed cells degenerated first, morphologically viable nuclei surrounded by disintegrated cytoplasmic debris were common (Fig. 5). Cells in denser areas showed atrophy of nuclear organelles. The nucleoli were shrunken and, like the reticulum and chromocentres, were less stained. Almost all surviving cells were akin to dying sarcoma cells in appearance. Viable cells endured until a 12th treatment but at this time cultures consisted almost wholly of intact hyperchromatic nuclei and amorphous dead acidophilic cytoplasmic debris. After further treatment the isolated nuclei were grossly degenerated in structure.

M/5,000 propiolactone administered daily in culture medium was progressively toxic and finally lethal to A2 fibrocytes. The effects of repeated treatments on interphase cells were individually slight but serially cumulative. The nuclear membrane gave considerable protection to nuclear structures but interphase nuclei were directly affected to some extent and also apparently indirectly through damage to cytoplasmic structures. Cell proliferation almost stopped after the third treatment. The population then stayed static for a few days before decreasing slowly. Cytoplasmic damage preceded degeneration of nuclei in the same cells. The nuclei continued to hypertrophy without degenerative structural changes until the cytoplasm was completely disorganized. The nuclear hypertrophy appeared to be an adaptive " orthogenetic " process of compensation for preceding cytoplasmic damage. However, with this dosage of lactone the treated fibrocytes failed to complete the enforced transformation and died before becoming malignant.

M/10,000 *propiolactone*

As expected, M/10,000 propiolactone solution was appreciably less acutely toxic than the previous drug concentration. Each application killed all cells in division, except prophases, at the time of treatment. The duration of the cytotoxic action following each application on mitotic cells decreased with drug concentration, and conversely the recovery periods between successive treatments lengthened. Even with M/100,000 lactone media cells in division at the time of application were killed. The cytological appearances also indicated that individual dividing cells, for example metaphases, died more quickly with stronger drug concentrations.

In general, the effects of each treatment with M/10,000 propiolactone were weaker and less prolonged. Cytotoxic effects and the counteracting nuclear hypertrophy also developed more slowly. The basophilic content of cytoplasm was reduced after two treatments. Nuclear hypertrophy was ambiguous after three but unmistakeable after four treatments. The cultures grew well until the 6th treatment. Thereafter proliferation decreased and was negligible after 10 treatments. Nuclear hypertrophy was preceded by decreased cytoplasmic basophilia and was manifest after 6 treatments.

By the 7th day primary nucleoli were enlarging at the expense of conjoined chromatin reticulum and chromocentres. At this time an increased basophilia of cytoplasm in healthy cells in conjunction with marked nuclear hypertrophy was observed. This phenomenon was never seen in cultures treated with M/5,000 propiolactone. The cytoplasm of cells in these latter cultures, with comparably hypertrophied nuclei, never regained their original content of basophilic material, although a partial restitution was achieved.

The morphological changes intensified during the 8th and 9th days of treatment, as the rate of proliferation was decreasing. After 9 treatments cytoplasmic damage was seen in peripheral cells and cells separated from local colonies. The greatly altered healthy basophilic cells, which showed no signs of cytoplasmic degeneration, could not be distinguished on morphological grounds from malignant sarcoma cells. They were classified as cytologically malignant (C.M.) cells.

The C.M. cells were larger than parent A2 fibrocytes, fusiform, with relatively large hyperchromatic nuclei and strongly basophilic cytoplasm. Their nuclear membranes were intensely stained. Primary nucleoli were much enlarged and irregular in shape. The reticular nucleolonema was usually visible in well developed basophilic matrix substance. Small chromocentres were deeply basophilic. Large chromocentres were also deeply stained but not greatly increased in number. Portions of chromatin reticulum and compound chromocentres formed small nucleolar-like structures. Around some of these complexes were deposits of basophilic background material similar to matrix substance of primary nucleoli. Small C.M. cells were also present. The process of nuclear hypertrophy in cultures treated with M/10,000 propiolactone followed the same pattern as that described for the M/5,000-treated cultures. In the present material, the nuclear organelles were markedly more basophilic.

A small proportion of C.M. cells were dividing. This was significant in view of the cessation of mitosis at an earlier stage in transformation. Many mitoses were abnormal but apart from typical lactone-induced aberrations, others such as tripolar mitoses, displaced and lagging chromosomes, were characteristic of malignant cells in general.

A 12th treatment produced no further development of the process of cell transformation but isolated cells were showing cytotoxic effects. A further treatment caused widespread damage to both C.M. and less altered cells. Thereafter, the cultures rapidly degenerated with subsequent treatments. Cytoplasmic degeneration tended to precede visible nuclear injury. However, this precession was much less evident than in cultures treated with M/5,000 lactone.

The effects of M/5,000, M/10,000 and M/20,000 solutions of propiolactone on collateral cultures of each set were not completely synchronized. The variation between two similarly treated slips fixed on the same day amounted to no more than the additional effects of a further treatment on one of the cultures. The optimal treatment with M/10,000 propiolactone for the induction of viable C.M. cells was about 11–12 successive daily applications. The transformation of cells treated with M/10,000 propiolactone was temporarily effective against the cytotoxic action of the drug as the induced malignant cells divided. With M/5,000 propiolactone treated cells died before completing the transformation to C.M. cells.

M/20,000 *propiolactone*

This concentration of the carcinogen had a short period of cytotoxic activity against dividing cells following each application of fresh solution. The effects of the initial treatment were negligible on interphase cells. The aberrations of mitotic cells were of the types described previously. The effects of three further treatments were similar and the growth of the cultures was not appreciaby retarded. A slight decrease in cytoplasmic basophilia appeared after five treatments. Most interphase cell nuclei at that time were unaltered but small numbers showed slight hypertrophy of nuclear organelles or slight diminution in nucleolar size and staining intensity. These latter regressive changes usually preceded nuclear hypertrophy.

After six treatments, nuclear hypertrophy was evident. Total cell population was increasing. The cytoplasm of some cells with enlarged nuclear structures stained more deeply than usual but the staining reaction was neutrophilic rather than basophilic.

After seven treatments darkly stained fusiform cells with hypertrophied nuclei were more abundant and their cytoplasm was more basophilic. The process of cell transformation towards the malignant state continued and C.M. cells were found after ten treatments. These cells increased in number for the next two days and were actively dividing. The optimal time for the induction of C.M. cells by M/20,000 propiolactone was about twelve days. These cells were typically sarcomatous and actively dividing.

Further treatments rapidly killed the C.M. and less altered cells. C.M. cells were more resistant than less altered fibrocytes to the cytotoxic action of carcinogenically optimal concentrations of propiolactone. Thus, C.M. cells, divided successfully after divisions in less altered cells had become very scarce. In the present experiments the C.M. cells developed while exposed to the carcinogen and may have inherited injured cell components.

If exposure to M/20,000 propiolactone was discontinued, C.M. cells rapidly proliferated. However, the stage of transformation at which replacement of lactone with normal medium was made was critical. It was essential that the carcinogenic process be completed but cultures not over-damaged by the carcinogen. With established A2 fibrocytes and a strongly growth-promoting medium. as used in these researches, the necessary conditions were difficult to attain, Present experiments indicate that cultures grown in less stimulating media are more easily controlled.

The C.M. cells cultivated in normal medium (Fig. 6, 7) varied widely in shape and size. The cytoplasm was strongly basophilic. Nuclear size was varied, but large relatively to the amount of cytoplasm. The mass and basophilia of primary nucleoli were much increased in comparison with untreated A2 cells. The nucleoli were usually irregular in form and rich in matrix material. The well-developed simple chromocentres showed increased basophilia. Compound chromocentres were abundant and enlarged. The markedly basophilic chromatin reticulum was thickened and often regionally hypertrophied. Surrogate nucleoli formed by associations of large chromocentres and hypertrophied portions of nuclear reticulum embedded in basophilic matrix substance were well developed. The total amount of nucleolar material, of primary and surrogate nucleoli, per nucleus was remarkedly great in comparison with untreated cells. The surrogate nucleoli were comparable morphologically to the much enlarged primary nucleoli. The nuclear membranes of these C.M. cells were markedly basophilic and sharply defined. They were often irregularly shaped with deep bays or more acute indentations. In most examples of the last, either a large compound chromocentre or nucleolus coincided with the apex of the concavity.

M/20,000 propiolactone was less toxic and less carcinogenic in terms of percentage conversion to C.M. cells than the M/10,000 concentrations. The carcinogenic process was less stimulated by the weaker dosage because of the slower rate at which cytoplasmic damage accumulated in cell lineages. There was a correlation between the cytotoxic and carcinogenic effects of propiolactone.

M/40,000 *propiolactone*

Cultures of A2 fibrocytes treated daily with M/40,000 propiolactone grew progressively for more than six weeks without deterioration. Interphase cells developed no observable lesions and showed no significant decrease in cytoplasmic basophilia. The deaths of dividing cells caused by each application of drug solution had no appreciable effect on growth of the cultures. Cells in prophase and most cells in other division phases were viable. " Exploded " metaphases were rare. Less acutely damaged but dead metaphases were commoner but still infrequent. Pyknotic anaphases and telephases were scanty.

Any cytoplasmic damage appeared to be adequately repaired before the next exposure to lactone solution as no evidence of degenerative changes was found. Slight hypertrophy of nuclear organelles began about the 5th day of treatment. This process was slowly developed until about the 11th day when primary nucleoli were slightly enlarged. Most simple chromocentres remained unchanged, and compound chromocentres were moderately larger. Nuclear hypertrophy did not progress beyond this stage. Altered cells never approached the C.M. stage. Surrogate nucleoli and hyperchromasia were never seen.

With one exception, no C.M. cells developed in any culture treated with M/40,000 propiolactone solutions. In this single example, the 16-day-old culture coverslip had been accidentally reversed in the roller-tube. The appearance of the culture indicated that the reversal had taken place about the 12th day of treatment. The appearances of cells on both sides of the slip were consistent with this inference. Depopulated areas on the originally upper surface of the coverslip had been recolonized by proliferating C.M. cells which were flattened and actively multiplying.

This phenomenon has been found before in studies on carcinogenesis *in vitro*. There appears to be a critical stage of about twelve consecutive treatments with a carcinogenically effective or sub-effective dosage of drug when reversal of the coverslip promotes the induction of C.M. cells. Negative results were obtained with cultures previously treaten for four weeks with M/40,000 propiolactone. The operative factor appeared to be a decreased growth rate of cells in the unfavourable locale between a coverslip and the wall of the roller-tube.

After 42 days on M/40,000 propiolactone (Fig. 8) the cultures were given normal medium daily for a week. Large numbers of these cells were inoculated subcutaneously in young adult rats. The treated animals gave the usual local inflammatory response but no tumours developed.

M/100,000 *propiolactone*

Treatments with this concentration had transient lethal effects on dividing cells. Very slight nuclear hypertrophy was observed. No C.M. cells were induced. Cells of cultures treate for 42 days later inoculated *in vivo* failed to produce tumours.

With the exception described above, M/40,000 and M/100,000 propiolactone solutions were carcinogenically inactive on A2 fibrocytes.

### DISCUSSION

A2 fibrocytes were serially treated with propiolactone at concentrations ranging from rapidly lethal (M/1,000) to indefinitely tolerated (M/40,000; M/100,000) dosages. Observations on cytological changes in the treated cells collectively suggested that transformation to the cytologically malignant state was the process by which they counteracted chronic damage caused by the carcinogen. The culmination of the adaptation by the cells to repeated cumulative injuries was the C.M. state itself. Cells did not further adapt in these experiments. Development of full cytological malignancy took place only at or near the maximal toxicity (M/10,000 ; M/20,000) tolerated long enough for transformation of the cells to be completed. With milder treatments (M/40,000 ; M/100,000) cells were not impelled to become malignant. With more severe treatment (M/5,000) cells failed to adapt completely and died prematurely.

There were certain differences between the effects on A2 and normal rat fibrocytes (Powell, 1966) of propiolactone. The established A2 fibrocytes grew continually at non-carcinogenic but not at carcinogenic drug concentrations. Normal fibrocytes on the other hand, became cytologically malignant when treated with concentrations which did not kill the cultures. In the former instance, the carcinogenic and cytotoxic effects almost coincided in magnitude, although cells became malignant before dying from further treatments. In the latter instance the carcinogenic process was completed in the absence of obvious general cytotoxicity.

M/40,000 propiolactone was the optimal concentration for induction of C.M. cells in cultures of normal fibrocytes. M/100,000 was also effective. Both these dosages were carcinogenically ineffective with A2 fibrocytes. Both were tolerated by both types of cells, apart from transient effects on mitotic cells. Stronger concentrations of the lactone were eventually lethal to each type of cell but A2 fibrocytes were more resistant and transformed to a greater degree with the intermediate drug concentrations tested. The relationship between general cytotoxicity and carcinogenic changes was clearer with A2 cells because of their greater resistance to the lactone.

These differences in response to propiolactone were attributable to differences in the growth rates of the two types of cells : A2 cells did not, unlike normal fibrocytes, grow more slowly after the first two weeks of cultivation, irrespective of experimental treatment.

Propiolactone is a potent but rapidly inactivated carcinogen (Dickens, 1962). Hexenolactone is less potent *in vivo* (Dickens and Jones, 1963) but less quickly inactivated in culture medium (unpublished work). The effects of this lactone on cultures of freshly derived normal rat fibrocytes were more akin to the effects on A2 fibrocytes than on normal fibrocytes of propiolactone. The accumulation of periodic serial injuries caused by a carcinogen appears to be greatly influenced by the balance reached between the damage done to a particular cell lineage with each application and the extent of the recovery achieved before the next treatment Growth rate, duration of the cytotoxic action after each drug application and the intrinsic cytotoxicity of the drug affect this balance.

The pattern of cytological changes undergone by A2 fibrocytes during the carcinogenic process was essentially the same as that seen in normal rat fibrocytes (Powell, 1966). The present observations are consistent with the interpretation of the malignant transformation then suggested.

In both types of fibrocytes, depletion of basophilic substances from cytoplasm was followed by hypertrophy of primary nucleoli and restoration of cytoplasmic basophilia. Primary nucleoli hypertrophied by the incorporation of thickening sections of chromatin network and enlarged chromocentres. Other chromocentres, not adjacent to primary nucleoli, and their associated sections of reticulum also hypertrophied to form nucleolus-like structures. These secondary nucleoli accumulated matrices of basophilic amorphous material.

Large chromocentres of interphase nuclei represent heterochromatic regions of chromosomes. The possible role of heterochromatin in the malignant process has been stressed by earlier workers. The chemical composition of nucleoli was reported by Caspersson and Schultz (1939) to be greatly influenced by heterochromatin. Schultz and Caspersson (1940) inferred that the RNA content of the nucleolus and cytoplasm was increased by the presence of the markedly heterochromatic Y chromosome in *Drosophila* cells. Caspersson and Santesson (1942) observed that actively dividing tumour cells were especially rich in heterochromatin. They stressed the role of heterochromatin in protein synthesis and the malignant process. The RNA content of tumour cells is markedly high (Leuchtenberger, Leuchtenberger and Klein, 1952 ; Laird, 1954 ; Rutman, Cantarow and Paschkis, 1954). Nucleoli are rich in RNA (Caspersson and Schultz, 1941 ; Brachet, 1941). Caspersson (1950) emphasized the relation of heterochromatin to protein synthesis and malignancy. Nucleoli are formed by specific heterochromatic regions, the nucleolar organizers, of particular chromosomes (Heitz, 1931 ; McClintock, 1934). Brachet (1957) concluded that there is a close relationship between heterochromatin and nucleoli.

Caspersson, Vogt-Köhne and Caspersson (1960) stressed the importance of disturbances in the nucleolus-cytoplasm system to an understanding of carcinogenesis. The present observations reinforce this view. The observed transformation of chromocentres and associated sections of chromatin network to nucleolus-like structures is further evidence of the close relation between heterochromatin and primary nucleoli. However, both the hypertrophy of primary nucleoli and the formation of the secondary nucleolar structures involved euchromatic sections of the chromatin network. Euchromatin is believed to carry the major specific genes (Brachet, 1957 ; Picken, 1960) and heterochromatin to be concerned with general gene functions. Thus the transformation of euchromatin into nucleolonema threads of primary and secondary nucleoli involved the loss of specific gene functions which were replaced by general synthetic functions. This interpretation of the cytological changes in fibrocytes treated with propiolactone affords a possible explanation of both the characteristic regressive changes in malignant cells (Haddow, 1955) and their rapid growth. The intranuclear distribution of hypertrophied organelles was not uniform from cell to cell except that portions of reticulum, and associated chromocentres, contiguous with primary nucleoli were normally affected. Different deletions of specific gene functions, resulting from modification of varying sections of euchromatin, could lead to variation among primary malignant cells developing together. The relationship of cytological changes during carcinogenesis to nucleolar functions and RNA of nucleoli and ribosomes have been discussed previously (Powell, 1966). The morphological changes in A2 fibrocytes during carcinogenesis described above conform with the interpretation then suggested.

### SUMMARY

1. The cytological effects of repeated treatments with the carcinogen, propiolactone, upon rat fibrocytes of an established strain are described.

2. Propiolactone induced the development of cytologically malignant cells.

3. Comparison of cultures treated with different concentrations of propiolactone showed a close correlation between cumulative toxicity and carcinogenicity.

4. Hypertrophy of primary nucleoli and the formation of nucleolus-like structures involving chromocentres and euchromatin, was observed in treated cells.

5. The significance of these observations to an understanding of carcinogenesis is discussed.

I am greatly indebted to Mr. G. A. Butcher and Mr. F. Butcher for their assistance with the tissue cultures and photomicrographs, and to Miss Sonia Baumann for the preparation of equipment and media. The expenses of this work were defrayed from a block grant by the British Empire Cancer Campaign for Research.

### REFERENCES

BRACHET, J.—(1941) *Enzymologia*, **10**, 87.
BRACHET, J.—(1957) ' Biochemical Cytology '. New York, (Academic Press), pp. 452, 113.
CASPERSSON, T.—(1950) ' Cell Growth and Cell Function '. New York (Norton).
CASPERSSON, T. AND SANTESSON, L.—(1942) *Acta Radiol.*, Suppl., 46.
CASPERSSON, T. AND SCHULTZ, J.—(1939) *Nature Lond.*, **143**, 602, 609.
CASPERSSON, T. AND SCHULTZ, J.—(1941) *Proc. natn. Acad. Sci. U.S.A.*, **26**, 507.
CASPERSSON, T., VOGT-KÖHNE, L. AND CASPERSSON, O.—(1960). In ' All Physiology of Neoplasia '. Edited by the Staff of M. D. Anderson Hospital, University of Texas, Austin. p. 269.
DICKENS, F.—(1962) In ' Cancer and Hormones '. University of Chicago, p. 107.
DICKENS, F. AND JONES, H. E. H.—(1961) *Br. J. Cancer*, **15**, 85.
DICKENS, F. AND JONES, H. E. H.—(1963) *Br. J. Cancer*, **17**, 100.
HADDOW, A.—(1955) *A. Rev. Biochem.*, **24**, 689.
HEITZ, E.—(1931) *Planta*, **12**, 775.
LAIRD, A. K.—(1954) *Expl Cell Res.*, **6**, 30.
LEUCHTENBERGER, C., LEUCHTENBERGER, R. AND KLEIN, E.—(1952) *Cancer Res.*, **12**, 480.
McCLINTOCK, B.—(1934) *Z. Zellforsch. mikrosk. Anat.*, **21**, 294.
PICKEN, L.—(1960) ' The Organization of Cells '. Oxford (Clarendon Press), p. 133.
POWELL, A. K.—(1966) *Nature, Lond.*, **209**, 77.
ROE, F. J. C. AND GLENDENNING, O. M.—(1956) *Br. J. Cancer*, **10**, 357.
RUTMAN, R. J., CANTAROW, A. AND PASCHKIS, K. E.—(1954) *Cancer Res.*, **14**, 3.
SCHULTZ, J. AND CASPERSSON, T.—(1940) *Proc. natn. Acad. Sci. U.S.A.*, **26**, 507.
WALPOLE, A. L., ROBERTS, D. C., ROSE, F. L., HENDRY, J. A. AND HOMER, R. F.—(1954) *Br. J. Pharmac. Chemother.*, **9**, 306.